# A Web-based database of genetic association studies in cutaneous melanoma enhanced with network-driven data exploration tools

**DOI:** 10.1093/database/bau101

**Published:** 2014-11-06

**Authors:** Emmanouil I. Athanasiadis, Kyriaki Antonopoulou, Foteini Chatzinasiou, Christina M. Lill, Marilena M. Bourdakou, Argiris Sakellariou, Katerina Kypreou, Irene Stefanaki, Evangelos Evangelou, John P.A. Ioannidis, Lars Bertram, Alexander J. Stratigos, George M. Spyrou

**Affiliations:** ^1^Center of Systems Biology, Biomedical Research Foundation, Academy of Athens, Soranou Ephessiou 4, 115 27 Athens, GR, Greece, ^2^Department of Dermatology, University of Athens, School of Medicine, Andreas Sygros Hospital, Ι. Dragoumi 5, 161 21 Athens, GR, Greece, ^3^Department of Vertebrate Genomics, Neuropsychiatric Genetics Group, Max Planck Institute for Molecular Genetics, Ihnestraße 63-73, 14195 Berlin, DE, Germany, ^4^Department of Neurology, Focus Program Translational Neuroscience, University Medical Center of the Johannes Gutenberg University Mainz, Mainz, DE, Germany, ^5^Department of Hygiene and Epidemiology, Clinical and Molecular Epidemiology Unit, School of Medicine, University of Ioannina, 451 10 Ioannina, GR, Greece, ^6^Department of Epidemiology and Biostatistics, Imperial College London, St Mary’s Campus, Norfolk Place, W2 1PG, London, UK, ^7^Department of Medicine Stanford Prevention Research Center, Stanford University School of Medicine, Stanford, CA, USA, ^8^Department of Health Research and Policy, Stanford Prevention Research Center, Stanford University School of Medicine, CA, USA, ^9^Department of Statistics, Stanford University School of Humanities and Sciences, Stanford, CA, USA and ^10^Department of Medicine, School of Public Health, Imperial College London, Sir Alexander Fleming Building, South Kensington Campus, London, UK

## Abstract

The publicly available online database *MelGene* provides a comprehensive, regularly updated, collection of data from genetic association studies in cutaneous melanoma (CM), including random-effects meta-analysis results of all eligible polymorphisms. The updated database version includes data from 192 publications with information on 1114 significantly associated polymorphisms across 280 genes, along with new front-end and back-end capabilities. Various types of relationships between data are calculated and visualized as networks. We constructed 13 different networks containing the polymorphisms and the genes included in *MelGene*. We explored the derived network representations under the following questions: (i) are there nodes that deserve consideration regarding their network connectivity characteristics? (ii) What is the relation of either the genome-wide or nominally significant CM polymorphisms/genes with the ones highlighted by the network representation? We show that our network approach using the *MelGene* data reveals connections between statistically significant genes/ polymorphisms and other genes/polymorphisms acting as ‘hubs’ in the reconstructed networks. To the best of our knowledge, this is the first database containing data from a comprehensive field synopsis and systematic meta-analyses of genetic polymorphisms in CM that provides user-friendly tools for in-depth molecular network visualization and exploration. The proposed network connections highlight potentially new loci requiring further investigation of their relation to melanoma risk.

Database URL: http://www.melgene.org.

## Introduction

Although a small fraction of mainly familial patients with cutaneous melanoma (CM) carry highly penetrant gene mutations (1–5), i.e. mutations in *CDKN2A* and *CDK4*, the more common sporadic form of CM is likely caused by the complex interplay of environmental and multiple genetic risk factors that exert moderate risk effects (1). Up to now, an increasing number of genetic association studies including candidate-gene and genome-wide association studies (GWAS) have been published that report on novel CM risk genes or attempt to validate previously reported genetic risk factors in CM (1). Owing to the enormous amount of partly contradictory information derived from those studies, the evaluation and interpretation of the genetic predisposition of CM is not a trivial task (6). To effectively collect and analyze all available information related to CM, we have implemented an online database named *MelGene* that provides a systematic and in-depth qualitative and quantitative catalog of genetic association studies in CM. Our database includes random-effects meta- analysis results of eligible polymorphisms that highlight the most compelling CM risk loci (7).

We conducted a systematic update in the *MelGene* database by including detailed summaries of all recently published association studies and by performing meta- analyses in all eligible polymorphisms that have been investigated in multiple studies to provide a summary effect for the association of each single-nucleotide polymorphism (SNP) to CM risk. The epidemiological validity of nominally significant meta-analysis results was assessed using the ‘Venice’ criteria suggested by the Human Genome Epidemiology Network (8). In this study, we present the new design of the front end and the back end of the database, providing the user with a more functional interface, easy-to-handle queries and embedded tools that facilitate the visualization and the exploration of the molecular relationship networks of putative genetic risk factors of CM. MelGene database provides a systematic and comprehensive overview of genetic association studies (both candidate-gene and GWAS) focusing exclusively on CM. Besides the embedded tools for data searching using keywords, *MelGene* database provides tools for automated meta- analysis of the collected data and allows for construction of networks using the available polymorphisms in the database. These features make *MelGene* database unique compared with databases such as GWAScentral (http://www.gwascentral.org/) or ‘The catalogue of Published Genome-Wide Association studies’ from the National Human Genome Research Institute that act as a compilation of summary-level findings and allow for searches between loci derived from GWA studies for various outcomes (http://www.genome.gov/gwastudies/).

## Material and methods

### Search strategy, data collection and meta-analysis

**Figure 1.**
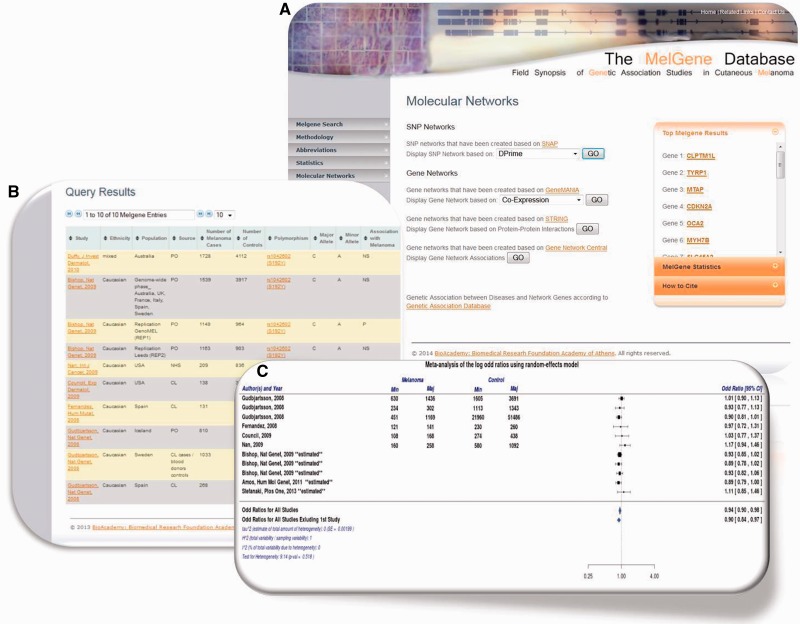
(**A**) Updated *MelGene* database search engine. Users are able to retrieve information available on *MelGene* based on keywords such as the gene name, polymorphism name, chromosome, first author of a publication, year of publication, ethnicity and the country of origin of study populations. (**B**) Polymorphism overview page and meta-analysis of polymorphism rs1042602 as an example. All publications that were included in *MelGene* and assessed rs1042602 in their data sets are listed in a sortable interactive table, and cross-links to the corresponding publications indexed in *PubMed* are provided in the database. (**C**) Forest plot of rs1042602 displays study-specific results as well as the summary *OR*, 95% CI and heterogeneity estimate.

For the continuous curation of the *MelGene* database, we performed systematic literature searches for peer-reviewed genetic association studies on CM using PubMed (http://www.ncbi.nlm.nih.gov/pubmed), the Human Genome and Epidemiology Network Navigator (http://hugenavigator.net) and the Melanoma Molecular Maps Project (http://www.mmmp.org/MMMP). The last search was conducted on 31 August 2013. The search strategy has been described in detail elsewhere (7). The current version of *MelGene* includes 192 publications that fulfilled our inclusion criteria [outlined in ref. (7)] and that report on 1114 polymorphisms across 280 genes. For each biallelic polymorphism included in the database with data of at least four independent case-control data sets (n = 79), a random- effects meta-analysis was calculated based on the DerSimonian and Laird model (7). In addition, between-study heterogeneity was quantified by the *I^2^* metric. Forest plots of the respective meta-analysis results were created using the ‘*metafor*’ package (9) in the R programming language (http://www.r-project.org/). Users are able to download those forest plots in high resolution (Figure 1B and C). The database curation is supported by an experienced team, which includes clinicians, biologists, bioinformaticians, biostatisticians and genetic epidemiologists.

### Database construction

The database scheme was created using *MySQL* (version 5.5.27, http://www.mysql.com/) and comprises the following fields: entry id, gene symbol, chromosome, location, study name consisting of first author name and year of publication, ethnicity, population and polymorphism name (where applicable, the official *NCBI*’s rs identifiers have been used, http://www.ncbi.nlm.nih.gov/snp/). In addition, fields were also supplemented by the following information where available: number of melanoma cases, number of controls, significance assessment by the authors of each publication, the minor and the major allele name based on genetic data available in the respective publication, minor–minor, minor–major and major–major genotype counts per study population, the allele frequency in CM cases as well as in control subjects, the additive odds ratio (*OR*) and 95% confidence interval (CI) limits if provided in the respective publication.

### Web application interface

The updated publicly available version of *MelGene* enables users to search the database based on a variety of parameters. More precisely, the database can be searched by gene name, polymorphism name, chromosome, name of the first author of a publication, the year of publication, the minimal number of cases per population, the geographical origin of study populations and by using a free text keyword search field. The updated Web-application search engine was implemented using *html*, *PHP* (http://php.net/) and *MySQL* (http://www.mysql.com/) queries (Figure 1A).

Moreover, for each polymorphism included in the *MelGene*, the updated database version provides links to several other genetic databases, and thus facilitates the retrieval of additional information on specific polymorphisms and genes of interest, i.e. *NCBI*’s *dbSNP* (http://www.ncbi.nlm.nih.gov/projects/SNP/), the International *HapMap* project’s database (http://hapmap.ncbi.nlm.nih.gov/), the Ensembl browser (www.ensembl.org), the *SNPedia* Web site (http://www.snpedia.com) and *GWAS* Central (https://www.gwascentral.org/).

### Embedded tools to visualize and explore molecular relationship networks

Pairs of polymorphisms and/or their corresponding genes that have been included in *MelGene* can be assessed for 13 different types of relationship. Each type of relationship drives to a different network representation where the nodes are either polymorphisms or genes and the edges represent the existence and the strength of the relationship between two nodes. Network representations of the *MelGene* data were created and uploaded on the *MelGene* Web server. The software used for the construction of each network and the type of the networks are provided in Table 1.

**Table 1.** Detailed list of all generated networks that are available in *MelGene*

AA	Software used to generate the network	Node type	Edge type
1	*GeneMANIA*	Gene	Co-expression
2	*GeneMANIA*	Gene	Protein–protein interactions
3	*GeneMANIA*	Gene	Genetic interaction
4	*GeneMANIA*	Gene	Shared protein domain
5	*GeneMANIA*	Gene	Co-localization
6	*GeneMANIA*	Gene	Pathway
7	*STRING*	Gene	Protein–protein interaction
8	*SABiosciences* Gene Network Central	Gene	Reported or predicted as CM (Literature)
9	*SNAP*	Polymorphism	Recombination rate
10	*SNAP*	Polymorphism	Genetic map distance
11	*SNAP*	Polymorphism	Genetic map position
12	*SNAP*	Polymorphism	DPrime
13	*SNAP*	Polymorphism	RSquared

The first column indicates the software used to generate each molecular relationship network. The second and third columns describe the type of nodes and associations (weights), respectively. *GeneMANIA URL*: http://www.genemania.org/; *STRING URL*: http://string-db.org/; *SABiosciences* Gene Network Central *URL*: http://www.sabiosciences.com/genenetwork/genenetworkcentral.php; *SNAP URL*: http://www.broadinstitute.org/mpg/snap/.

The list of all 280 genes included in *MelGene* has been used as input to *Cytoscape* (http://www.cytoscape.org/) (10), an open-source software for integration, visualization and analysis of biological networks. Specifically, the *GeneMANIA* plug-in (http://www.genemania.org/) (11) of *Cytoscape* has been used. This plug-in retrieves a list of genes that are related to the input genes based on a large set of functional data, including (i) co-expression, where two genes are linked if their expression levels are similar across conditions in a gene expression study, (ii) protein–protein interactions, (iii) genetic interaction, where two genes are functionally linked if perturbations of one gene has an impact to the second, (iv) shared protein domains, where two genes are linked if they have the same protein domain, (v) co-localization, where genes are linked if they are expressed in the same tissue and (vi) pathways, where two genes are linked if they are part of the same pathway [see (11) for more details]. With the use of *GeneMANIA*, new members of a pathway or a complex interaction can be highlighted.

Moreover, two additional relationship networks have been constructed. The first network was a protein–protein interaction network created by means of the *STRING* database (http://string-db.org/) (12), whereas a second network was created based on the question of whether the input genes have been reported or predicted as being involved in CM pathophysiology in the literature, according to *SABiosciences* Gene Network Central (13).

In addition, all 1114 polymorphisms included in *MelGene* were submitted to *SNAP* (http://www.broad institute.org/mpg/snap/) (14), yielding another six sets of networks. *SNAP* provides pair-wise SNP’s calculations of input against the ‘1000 Genome Pilot 1’ SNP data set based on phased genotype data from the International *HapMap* Project (http://hapmap.ncbi.nlm.nih.gov/) (15). Using the *SNAP* tool with the 1114 SNPs, five additional molecular relationship networks were created using as network relationship measure (i) the recombination rate in centimorgans per million bases, (ii) the genetic map distance (that is, the distance from the query SNP to the proxy SNP in centimorgans), (iii) the genetic map position (that is, the position of the SNP on the genetic map for this chromosome in centimorgans), (iv) *D’* [measure of linkage disequilibrium (*LD*) normalized to allele frequency] and (v) the *r*^2^ correlation coefficient [see (14) for more details].

**Figure 2.**
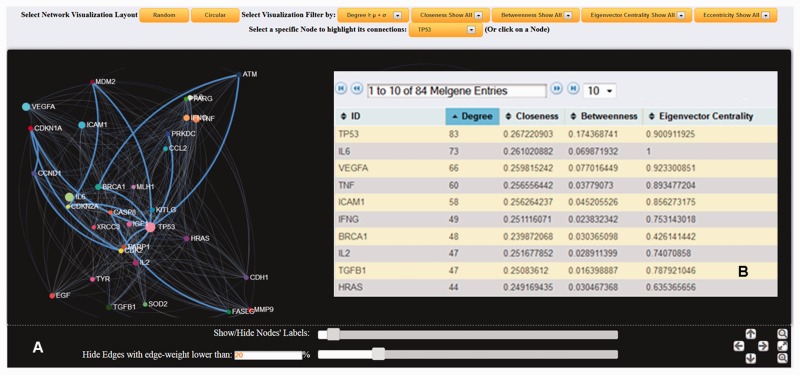
(**A**) Dynamic gene interaction network based on protein–protein interactions using the gene *TP53* as an example. First neighborhood interactions of *TP53* are highlighted against the entire protein–protein interaction network. In the present example, edges (interconnections) with weight <20% of the maximum weight value were omitted, and node (gene) labels with >10 interactions are displayed. (**B**) List of the calculated node-specific network features corresponding to node *TP53* is provided.

**Figure 3.**
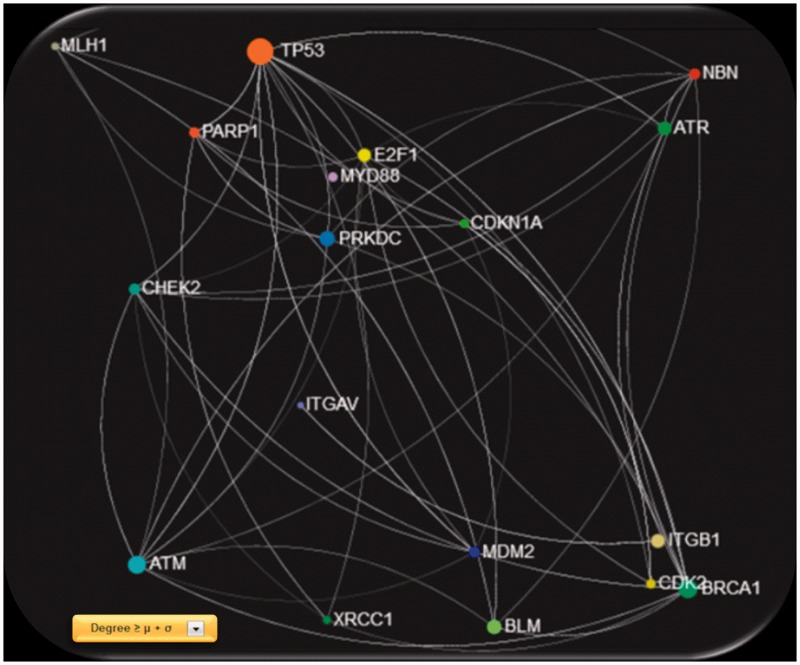
Screenshot of the gene network created using *GeneMANIA* physical interactions, filtered with degree ≥ μ + σ, were μ and σ are the mean value and standard deviation of the entire degree feature vector, respectively.

Both SNP and gene interaction network visualization tools were implemented in *MelGene* by means of the *jQuery* (http://jquery.com/) and ‘*sigma.js*’ (http://sigmajs.org/) *JavaScript* libraries. Figure 2 shows an example of an interaction network based on genes’ co-expression properties. Networks can be created on a random or circular layout. The node’s size is proportional to the number of first neighbors that are interconnected to the specific node. A larger size signifies a larger number of interconnections. Users of *MelGene* can locate and highlight a specific node interactively, either by using a drop-down box or by clicking on the node. The color and the weight of the edge between two interconnected nodes are an indicator of how closely these two nodes are related. White color corresponds to low, whereas cyan to high linkage weight. In addition, when a node is selected, node-specific network features [see (16) for detailed description] of their first neighbors are also provided, including the degree (i.e. number of adjacent edges), closeness (which measures how many steps are required to access every other node from a given node), betweenness (i.e. the shortest path from one node to another), and eigenvector centrality (a natural extension of degree centrality measuring the importance of a node if it is linked to by other important nodes). All the aforementioned features were *a* *priori* calculated by means of the ‘*igraph*’ (http://cran.r-project.org/web/packages/igraph/) (16) *R* package tool (http://www.r-project.org/) applied to each created relationship network. For each network, users are also able to apply network visualization filtering based on the mean value and the standard deviation calculated by the entire selected feature vector. This filtering can be performed either locally by selecting a node or globally to the entire network and can reveal ‘hub’ nodes in a more robust approach (Figure 3).

## Results and discussion

After a comprehensive data collection and systematic meta-analyses of CM association studies in *MelGene*, 20 genes showed genome-wide significant (*P* < 5 × 10^−^^8^) evidence for association with CM risk (*MelGene top genes*, Table 2). In addition, summary *OR*s and 95% CIs, heterogeneity as well as summary estimates on exclusion of the first published study were calculated and the respective plots have been made publicly available in the database. Moreover, the new network visualization tools developed in the updated *MelGene* database provides additional information about interconnections among genes or SNPs that may be indirectly related to CM. In the following result sections, we depict some genes and polymorphisms highlighted from the network-driven analyses that were performed in the *MelGene* environment.

**Table 2:** .Ranked list of the most significantly associated CM genes according to *MelGene*.

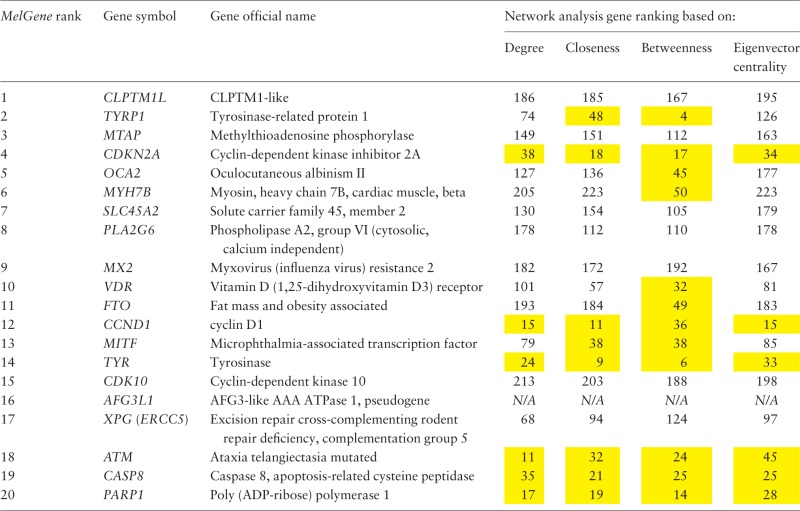

Network features were calculated on the relationship network produced by the *STRING* platform. In the present example, we have performed gene ranking for each network feature (of note, the pseudogene *AFG3L1* was not found in the STRING platform and thus excluded from this list). The genes that rank among the top 50 of the respective network features are highlighted in yellow.

**Figure 4.**
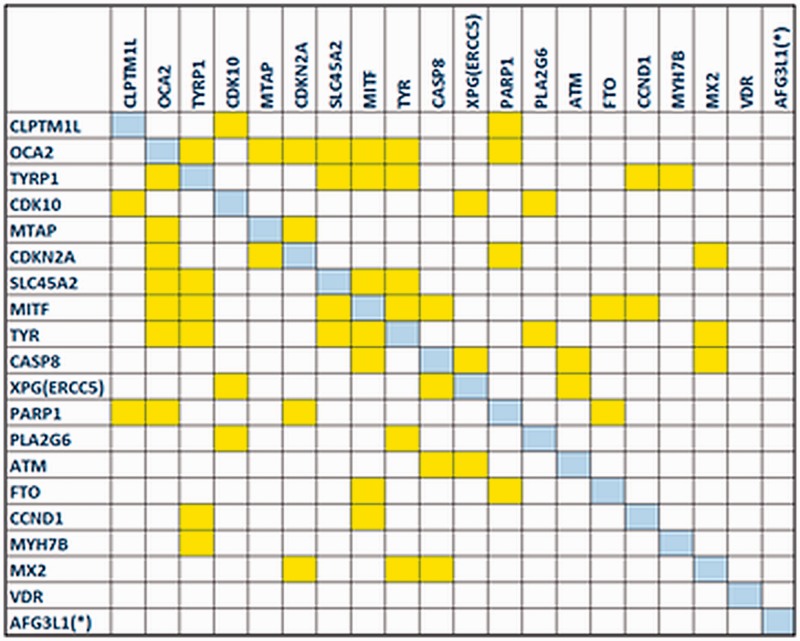
The co-expression matrix for the *MelGene top genes* that showed genome-wide significant (*P* < 5x10^−8^) evidence for association with CM risk.

The gene co-expression network reconstructed by the *GeneMANIA* plug-in revealed that the majority of *MelGene top genes* (18 of 20) are interconnected with at least one edge, presenting a high level of co-expression between these genes. In Figure 4, we see the corresponding co-expression matrix for these genes.

Furthermore, in the same gene co-expression network, we investigated which genes act as ‘hubs’ and assessed their relationship with the *MelGene top genes*. We calculated four network properties per node: degree, closeness, betweenness and eigenvector centrality. In the sequel, we calculated their average values (μ) and the corresponding standard deviations (σ). For each property, we considered as ‘hubs’ the genes that present a property value above μ + σ, and finally we merged the ‘hub genes’ from the four properties in one list. Afterward, we examined whether genes from Table 2 are ‘hub genes’ or are they connected with other ‘hub genes’. We found that the genes *MITF*, *TYR* and *PARP1* of Table 2 are ‘hub genes’ and that the genes *CDKN2A*, *OCA2*, *MX2*, *VDR*, *MITF* and *CASP8* are interconnected with >10 ‘hub genes’ as shown in Table 3.

**Table 3.** Ranked list of the most significantly associated CM genes according to *MelGene* along with the number and the names of the interconnected ‘hub genes’ in the corresponding co-expression network

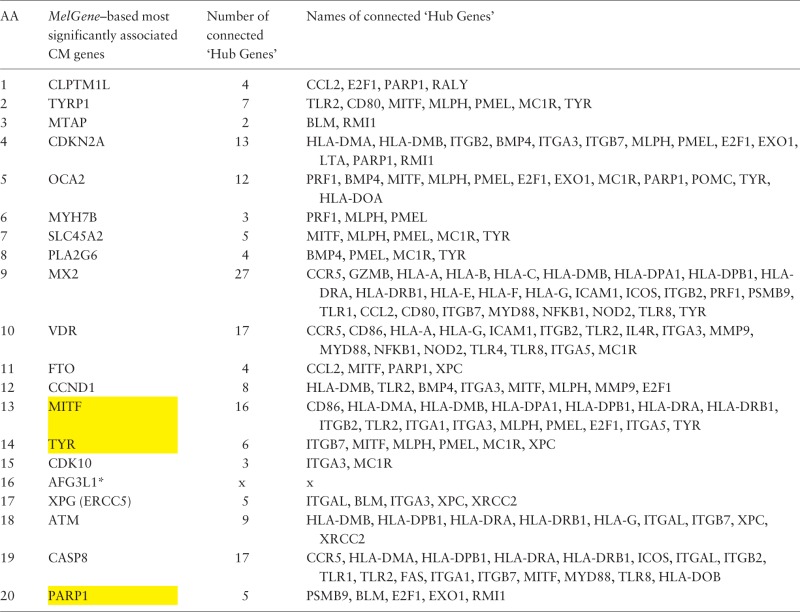

Yellow highlighted are those genes that act also as ‘hubs’. * AFG3L1 is reported as pseudogene.

**Figure 5.**
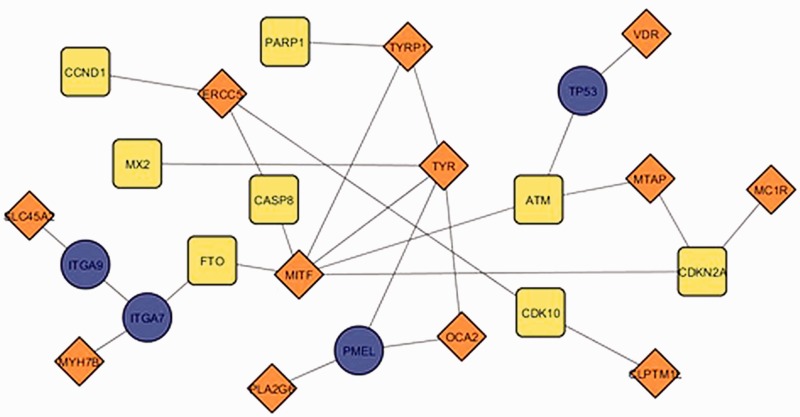
The fully connected subnetwork of the genes from two successive field synopses of *MelGene* that showed genome-wide significant evidence for association with CM risk. Orange, blue and yellow corresponding to the significant genes of the previous *MelGene* version, the significant genes of the current *MelGene* analysis and the genes required for a fully connected subnetwork, respectively.

The current update of *MelGene* database includes 20 genes that showed genome-wide significant (*P* < 5 × 10^−^^8^) evidence for association with CM risk compared with 12 genes described in the previous field synopsis (7). Investigating the relationship between the previous and the current *MelGene top gene* list, we mapped the genes on the network constructed by GeneMANIA with all the available relationship types (co-expression, co-localization, genetic interactions, physical interactions, pathways and share protein domain). The fully connected (i.e. nodes without a connection are absent) subnetwork of these genes is shown in Figure 5. The significant genes of the previous update are visualized with orange diamonds; the significant genes of the current field-synopsis are visualized in yellow whereas the genes required for a fully connected subnetwork are highlighted in blue (*TP53*, *PMEL*, *ITGA7* and *ITGA9*; Figure 5).

We found that the genes *TYRP1*, *TYR*, *MITF* and *ERCC5* displayed in Figure 5 are ‘important connectors’ in the network as they interact with 3, 5, 6 and 3 ‘significant’ genes that were significant using random-effects meta- analysis. Investigating these genes further, we found that they have been implicated in CM in the literature and/or by several other databases. More specifically, p53 has been suggested to be a major player suppressing progression from nevi to melanoma (17, 18). Aside from its role in pigmentation, PMEL (SILV) encodes antigenic epitopes that are recognized by multiple melanoma diagnostic antibodies including HMB-45, currently one of the most commonly used melanocytic markers for clinical melanoma diagnosis in humans (19). Furthermore, for *PMEL*, MalaCards, a database of human maladies and their annotations (www.malacards.org) (20), as well as the DISEASES database (Disease-gene associations mined from literature) developed by the University of Copenhagen (http://diseases.jensenlab.org) rank CM as the first disease related for *PMEL*. Finally, proteins encoded by ITGA7 and ITGA9 genes belong to the integrin alpha chain family. Changes in integrins expression have been reported during the malignant progression of many tumors, and much evidence exists implicating their involvement in CM metastasis (21). Malacards also implicates *ITGA7* and *ITGA9* in CM (eighth and third rank of gene-related diseases, respectively).

Another important gene in the co-expression network is *MLANA*. *MLANA*, a key network element, was found to be co-expressed with 8 (*TYRP1*, *CDKN2A*, *OCA2*, *SLC45A2*, *PLA2G6*, *MX2*, *MITF* and *TYR*) from the 20 *MelGene top genes* of the current *MelGene* gene list. CM is ranking first among the diseases related to *MLANA* according to the *MalaCards* and *DISEASES* databases, but its role as a risk gene for CM in *MelGene* currently remains unclear due to lack of sufficient association data.

In addition, *ITGB2* was found to act as a ‘hub gene’ by all centrality measures and is co-expressed with 5 (*CDKN2A*, *MX2*, *VDR*, *MITF* and *CASP8*) from the 20 *MelGene top genes*. Moreover, CM is ranking sixth among the diseases related to *ITGB2* according to Malacards.

Finally, regarding *HLA-A*, CM is ranking third among the related diseases according to MalaCards and the DISEASES database. *HLA-A* is co-expressed with 2 (*MX2* and *VDR*) of the 20 *MelGene top genes* and it acts as a ‘hub gene’ according to three important centrality metrics (Degree, Closeness and Eigenvector centrality).

In a similar way, we examined the networks of single-nucleotide polymorphisms (SNPs) based on their DPrime value with proxy SNPs. Specifically, we found the SNPs acting as ‘hubs’ in these networks and investigated their relationship with the top 20 SNPs that correspond to the 20 *MelGene top genes*. Again, we calculated the four network centrality measures. The SNPs that present at least one network property value above μ + σ are considered to act as ‘hub’ SNPs. We merged the ‘hub’ SNPs derived from the four different metrics resulting in 607 unique ‘hub’ SNPs, eight of which belong to the top 20 SNPs of *MelGene* (rs401681, rs1408799, rs2218220, rs6001027, rs11263498, rs1393350, rs1801516 and rs3219090—Table 4, highlighted in yellow). The SNP4Disease database (http://snp4disease.mpi-bn.mpg. de/), which was developed by the Max Planck Institute for Heart and Lung Research and provides information on diseases linked to SNPs by literature-mining techniques from various sources, was used to find which of the 607 ‘hub’ SNPs have been implicated in CM. In all, 72 ‘hub’ SNPs have been linked to CM, seven of them belong to the top 20 SNPs. Furthermore, we examined whether the top 20 SNPs are connected with ‘hub’ SNPs in the DPrime network (rs14961795, located in *MITF*, was not found in SNP DPrime network and thus excluded from this list). Five of the top 20 SNPs interact with at least one ‘hub’ SNP (rs408799, rs2218220, rs6001027, rs1126349 and rs1393350) as shown in Table 4.

**Table 4.** Ranked list of the most significantly associated CM SNPs according to *MelGene* along with the number and the names of the interconnected ‘hub SNPs’ in the corresponding DPrime SNP network

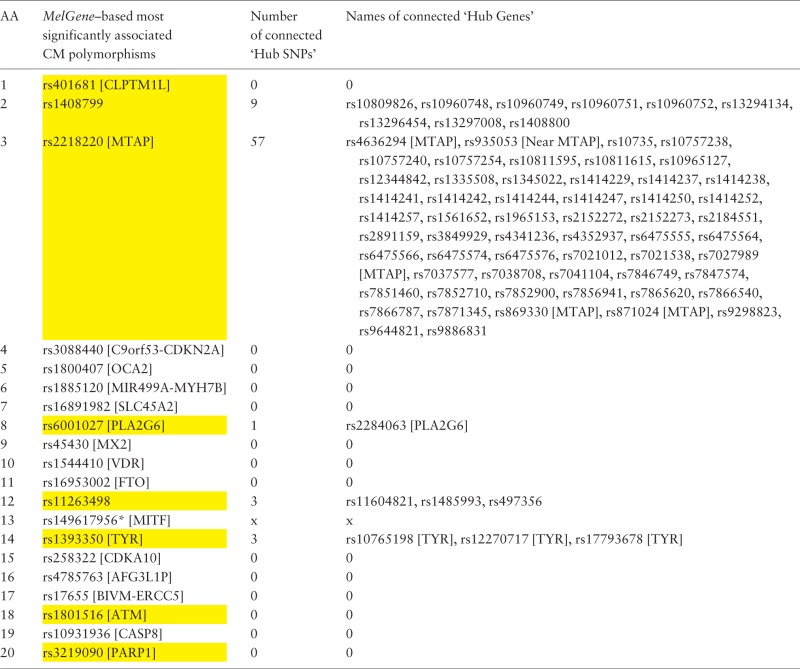

Yellow highlighted are those SNPs that act also as ‘hubs’. * Indicates that the specific SNP does not exist on the DPrime SNP network.

**Figure 6.**
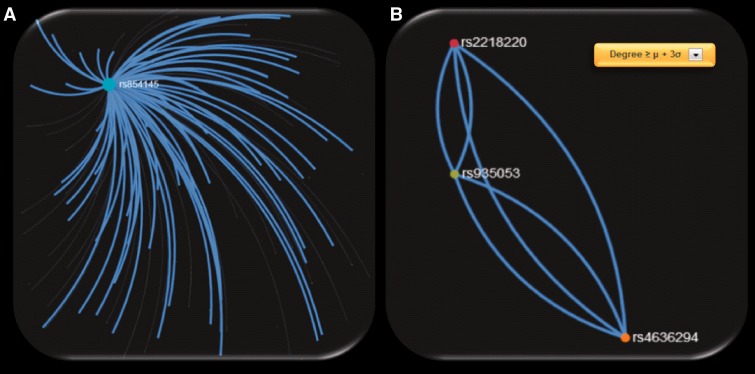
Screenshots of SNP networks created based on the *DPrime* SNP network of (**A**) rs854145 with no visualization filtering, (**B**) rs935053 with Degree ≥ μ + 3σ.

When exploring the 72 ‘hub’ SNPs that were implicated in CM by the SNP4Disease database in the polymorphism networks based on SNP DPrime values, we observed that four SNPs [rs2218220 (located in *MTAP*), rs4636294 (located in *MTAP*), rs854145 (located in *GRM1*) and rs935053 (located Near *MTAP*)] acted as ‘hubs’ based on three centrality measures (degree, closeness and betweenness centrality). We observed that one of these SNPs, i.e. rs854145 in *GRM1*, is the most central polymorphism (see Figure 6A). On the other hand, rs2218220, which is located in *MTAP*, belongs to the 20 most significant SNPs in the *MelGene* meta-analysis results. Finally, rs4636294 and rs935053 were also found as ‘hub’ SNPs from the three centrality metrics. As shown in Figure 6B, they are also highly intercorrelated with rs2218220.

To highlight the most important ‘hub’ SNPs, we applied the same procedure but we considered as ‘hubs’ the SNPs that present at least one centrality measure above μ + 3σ. We obtained 256 ‘hub’ SNPs for the 4 centrality measures of which 2 are from the 20 most significant SNPs in the *MelGene* meta-analysis results (rs11263498 [located in *CCND1*] and rs2218220 [located in *MTAP*]). From the 256 ‘hub’ SNPs, 21 are reported in the ‘SNP4Disease’ database in relation to CM. Strikingly, the 21 SNPs derived with more rigorous centrality filtering (at least one centrality measure above μ + 3σ) included again the same central polymorphisms (rs854145, rs2218220, rs4636294 and rs935053) found previously in the 72 ‘hub’ SNPs that were implicated in CM by the SNP4Disease database with at least one centrality measure above μ + σ.

In summary, by using several pipelines network-driven data exploration tools implemented in *MelGene*, we have identified seven genes from the *MelGene* database (none of which had sufficient association data to perform a meta-analysis in *MelGene*) and four polymorphisms (of which three have not been meta-analyzed in *MelGene* because of lack of association data) that may play a potential role in CM pathophysiology (Table 5).

**Table 5.** List of genes and polymorphisms highlighted through network-driven analysis based on *MelGene* data that have not been meta-analyzed in *MelGene* beacuse of lack of sufficient data

Gene	SNP	Study	Ethnicity	Population	Source	Number of melanoma cases	Number of controls	Major allele	Minor allele	Association with melanoma
PMEL (SILV)	rs1052206	Fernandez, Hum. Mutat., 2008	Caucasian	Spain	CL	131	245	T	C	NS
	rs1052165	Fernandez, Hum. Mutat., 2008	Caucasian	Spain	CL	131	245	C	T	NS
	rs2069391	Fernandez, Hum. Mutat., 2008	Caucasian	Spain	CL	131	245	C	T	NS
MLANA	rs2233178 (H17)	Fernandez, Exp. Dermatol., 2009	Caucasian	Spain	CL	205	245	C	T	NS
TP53	rs1042522	Nan, Br. J. Dermatol., 2008	mixed	USA	NHS	211	850	C	G	P
		Povey, Carcinogenesis, 2007	Caucasian	UK	CL cases / PO controls	538	425	C	G	NS
		Stefanaki, Br. J. Dermatol., 2007	Caucasian	Greece	CL	107	145	C	G	P
		Han, Mol. Carcinog., 2006	mixed	USA	NHS	NA	NA	NA	NA	NA
		Gwosdz, Int. J. Cancer, 2006	Caucasian	Germany	CL cases / blood donors controls	49	193	C	G	P
		Shen, J. Invest. Dermatol., 2003	Caucasian	USA	CL	NA	NA	NA	NA	NA
		Bastiaens, Mol. Carcinog., 2001	Caucasian	The Netherlands	CL	120	157	C	G	NS
		Capasso, J. Hum. Genet., 2010	Caucasian	Italy	CL	240	284	C	G	NS
		Li, J. Invest. Dermatol., 2008	Caucasian	USA	CL	805	838	C	G	P
ITGA7	rs1800974	Lenci, Mutagenesis, 2012	Caucasian	Germany	CL	757	736	G	A	NS
ITGA9	rs267561	Lenci, Mutagenesis, 2012	Caucasian	Germany	CL	757	736	G	A	NS
ITGB2										
HLA-A	HLA-A*01, HLA-A*02, HLA-A*03, HLA-A*11, HLA-A*23, HLA-A*24, HLA-A*25, HLA-A*26, HLA-A*28, HLA-A*29, HLA-A*30, HLA-A*31, HLA-A*32, HLA-A*33, HLA-A*34, HLA-A*66, HLA-A*00	Campillo, Immunogenetics, 2006	Caucasian	Spain	CL cases / PO controls	174	227	NA	NA	NS
	HLA-A*01, HLA-A*02, HLA-A*03, HLA-A*11, HLA-A*23, HLA-A*24, HLA-A*25, HLA-A*26, HLA-A*29, HLA-A*30, HLA-A*31, HLA-A*32, HLA-A*33, HLA-A*36, HLA-A*68, HLA-A*69, HLA-A*80	Naumova, Cancer Immunol Immunother., 2005	Caucasian	Bulgaria	PO	50	54	NA	NA	NS
	HLA-A*01	Luongo, Tissue Antigens, 2004	Caucasian	Italy	CL cases / PO controls	382	203	NA	NA	NS
GRM1	rs854145	Ortiz, Eur. J. Hum. Genet., 2007	Caucasian	Spain	CL	250	329	T	C	NS
MTAP	rs4636294	Bishop, Nat. Genet., 2009	Caucasian	Genome-wide phase_ Australia, UK, France, Italy, Spain, Sweden.	PO	1539	3917	A	G	P
		Bishop, Nat. Genet., 2009	Caucasian	Replication GenoMEL (REP1)	PO	NA	NA	NA	NA	NA
		Bishop, Nat. Genet., 2009	Caucasian	Replication Leeds (REP2)	PO	NA	NA	NA	NA	NA
			Caucasian	Australia_Q-MEGA	PO	1734	1811	A	G	P
			Caucasian	UK_Leeds2	PO	1397	2465	A	G	P
MTAP region			Caucasian	Greece	CL	284	284	A	G	NA
GWA_rs935053	rs935053	Bishop, Nat. Genet., 2009	Caucasian	Genome-wide phase_ Australia, UK, France, Italy, Spain, Sweden.	PO	1539	3917	G	A	NS
		Bishop, Nat. Genet., 2009	Caucasian	Replication GenoMEL (REP1)	PO	1149	964	G	A	NA
		Bishop, Nat. Genet., 2009	Caucasian	Replication Leeds (REP2)	PO	1163	903	G	A	NA
Near MTAP		Amos, Hum. Mol. Genet., 2011	Caucasian	USA_MD Anderson Cancer Center	CL	1804	1026	G	A	P

*Source*: ‘CL’ (clinic based), ‘PO’ (population based), ‘NHS’ (Nurses Health Study), ‘HPFS’ (Health Professionals Follow-up Study).

Association with Melanoma: Overall conclusion reached by authors of the original publication (‘P’ indicates significant (*P* < 0.05) association in at least one of the performed analyses, and ‘NS’ indicates nonsignificant association); results obtained in duplicate or largely overlapping samples are listed as ‘NA’.

## Conclusion and future work

We present here the new re-designed version of the *MelGene* database empowered with network analysis tools that has led to the identification of known and several new promising genes implicated in melanoma pathophysiology. *MelGene* serves as a comprehensive reference repository for genetic association data in CM and can provide further insights in the predisposition of CM by systems biology approaches.

In the future, it is planned to further expanding the *MelGene* database by curating our data set with new small- and large-scale genetic association studies in regular time intervals. In addition, our database can be expanded so as to include and integrate more data in the future including somatic mutations in melanoma from publicly available databases such as the COSMIC database (22), as well as more and of different types of pre-compiled relationship networks.

## Funding

This research has been co-financed by the European Union (European Social Fund – ESF) and Greek national funds through the Operational Program ‘Education and Lifelong Learning’ of the National Strategic Reference Framework (NSRF) - Research Funding Program: Aristeia I - 1094. The funders had no role in study design, data collection and analysis, decision to publish, or preparation of the manuscript.

*Conflict of interest*. None declared.
